# A Randomized, Double-blind, Placebo-controlled Trial of Celecoxib Augmentation of Sertraline in Treatment of Drug-naive Depressed Women: A Pilot Study

**Published:** 2015

**Authors:** Marzieh Majd, Farshad Hashemian, Seyed Mohammad Hosseini, Maryam Vahdat Shariatpanahi, Ali Sharifi

**Affiliations:** a *Department of Clinical Pharmacy, Pharmaceutical Sciences Branch, Islamic Azad University, Tehran, Iran.*; b *Department of Psychiatry, Hamadan University of Medical Sciences, Tehran, Iran.*; c *Department of Psychiatry, Tehran Medical Branch, Islamic Azad University, Tehran, Iran.*; d *Research Center for Intelligent Signal Processing (RCISP), Tehran, Iran.*

**Keywords:** Celecoxib, First episode of major depression, Inflammatory cytokines

## Abstract

This study was designed to examine the antidepressant effect of celecoxib (200 mg/day) augmentation of sertraline in the treatment of female patients with first episode of major depression over 8 weeks of therapy.

Thirty female outpatients diagnosed with first episode of major depression, were recruited for this study. Participants were randomly assigned into two equal groups receiving either sertraline plus celecoxib 100 mg twice daily or sertraline plus placebo twice daily. Patients were assessed by Hamilton Depression and Anxiety Rating Scale at baseline, week 4 and week 8 of treatment.

Both treatment groups showed notable improvement in their symptoms from baseline; however, celecoxib group showed greater decrease in Hamilton Depression Scores compared to the placebo group after four weeks of treatment. Response rates were also found to be significantly higher in the celecoxib group compared to the placebo group over 4 weeks. Nevertheless, the mentioned differences between two groups were not significant at the end of week 8. Also, remission rate was remarkably higher in celecoxib group in comparison with placebo at the end point.

The results suggested that celecoxib may hasten the onset of therapeutic action of sertraline and increase response and remission rate in depressive disorders.

## Introduction

Depression is a common and potentially debilitating mental disorder which is estimated to have a two-fold higher incidence rate in women than in men. Despite substantial efforts to understand the nature of disease, the exact underlying mechanism remains vague. 

Drug intervention is the mainstay of treatment for major depression, although it is recognized that antidepressants have delayed onset of therapeutic effects and optimal therapeutic action is achieved after a lapse of at least four weeks (1). Since most antidepressant medications were believed to work through monoamine reuptake inhibition, other mechanisms have been proposed to play a role in the pathology of depression as well (2-7). 

A large number of evidences do suggests that inflammatory process and immune responses are involved in the pathophysiology of depression. Both animal studies and human clinical trials showed that endotoxins exposure and proinflammatory cytokines administration may induce depressive-like symptoms (8-13). Notably, up to half of the patients receiving interferon-α therapy may experience depressive symptoms during treatment course which may result in discontinuation of their therapies (14-16). 

Also, elevated levels of pro-inflammatory cytokines, such as interleukin (IL)-6, have been demonstrated in plasma and cerebrospinal fluid of depressed subjects (17-19). Moreover, it was suggested that peripherally released cytokines may enter the brain and exert their effects interfering with normal neural structure and activity (14, 20, 21).

Depression appears to be associated with cyclo-oxygenase (COX)-2 overexpression in the rat brain (22). COX-2, an inducible COX isoform, plays a dominant role in the inflammatory process. Thus, COX-2 inhibitors such as celecoxib, are proposed to have antidepressant properties. The therapeutic effect of celecoxib was indicated in animal models of depression (23). Beneficial effect of celecoxib adjunctive treatment was reported in psychiatric conditions, such as bipolar disorder and schizophrenia as well (24-26). Furthermore, two studies showed some favorable effects of celecoxib in major depression when added to reboxetine and fluoxetine treatment (27, 28).

While depression itself is considered a heterogeneous disorder, the probable role of immune system in the pathogenesis of the disorder can complicate the matter further. Meanwhile, several studies have shown that antidepressants exert immunomodulatory properties that may affect the human immune system and may partly contribute to their efficacy (29-32). It must be noted that immune response profoundly varies between different genders due to differences in sex hormones (33). To the best of our knowledge, there have been no controlled studies investigating the beneficial additive effect of celecoxib in the treatment-naïve depressed women. Bearing in mind, we are proposing it is important to only consider female patients whose immune system is not already affected by previous administration of antidepressants.

The present research is the first clinical study which was designed to examine the antidepressant properties of celecoxib (200 mg) while added to sertraline in the treatment of drug-naïve women with first episode of major depression.

## Experimental

*Study design*

This study was a prospective, randomized, double-blind, parallel-group, placebo-controlled pilot study conducted at two psychiatric centers in Iran. 

The trial was registered in Iranian Registry of Clinical Trials (IRCT registration number: IRCT201009043106N3). The study was approved by Ethics Committee of Islamic Azad University, Pharmaceutical Sciences Branch (No: 4114). All Women participated in the study were informed of the study procedure and signed written consent forms. 

*Participants*

Female patients diagnosed with major depression according to Diagnostic and Statistical Manual for Mental Disorders, fourth Edition Text revision (DSM-IV-TR) criteria were enrolled in the study. Thirty patients (15 in each arm) met all the following inclusion criteria: Female gender, first episode of unipolar major depressive disorder based on SCID (Structured Clinical Interview for DSM-IV) diagnostic instrument, no history of antidepressant use, age between 18-50 years, Hamilton Depression Rating Scale (17 items) Score ranging from 18 to 36 at baseline.

Participants were excluded from the study if they met the following criteria: History of other DSM-IV axis I psychiatric disorders, history of substance abuse, personality disorders, significant suicidal thoughts, hepatic disease, any cardiovascular disorders, gastrointestinal ulcer, inflammatory bowel disease, past history of GI bleeding (not within 2 years), Planning for pregnancy/lactation, hypersensitivity to celecoxib and sulfonamides.

To increase homogeneity of the study population, the present study was carried out among drug-naïve women.

*Randomization and allocation concealment*

In this study a table was prepared based on random-number table by a person who was blinded to the study. Then, this data was put inside a sealed envelope and was given to a pharmacist who was blinded to the study as well. The pharmacist coded the drugs according to the prepared table and decoded them at the end of the study (after statistical analysis). The table was prepared before the initiation of patients’ recruitment so that the Hamilton depression scale scores could not bias patients’ recruitment. 

*Intervention and clinical assessment*

Eligible patients were randomly assigned into two equal groups receiving either sertraline plus celecoxib 100 mg/twice daily or sertraline plus placebo twice daily for 8 weeks. Celecoxib and placebo capsules were identical in appearances. All patients had been instructed to take 25 mg/day of sertraline for the first 3 days, then the dose was increased to 50 mg daily. Patients were re-evaluated after 4 weeks and an increase in sertraline dosage to 100 mg/day was done if it was needed. Severity of depression and treatment response were measured by Hamilton Depression Rating Scale (HAM-D). The Hamilton Anxiety Rating Scale (HAM-A) was used to quantify the severity of patient's anxiety. All patients were assessed by a medical professional at baseline, week 4, and 8 of treatment. It should be noted that patients’ conditions were assessed each week via phone to check adherence and side effects. 

*Efficacy measures*

The mean change in HAM-D total score from baseline was used to evaluate the efficacy of celecoxib augmentation in depression symptoms (primary outcome). Furthermore, treatment response which is defined as ≥ 50% drop in HAM-D total score (1), was measured at week 4 and 8 of study. Moreover, remission defined as HAM-D total score ≤ 7 (1), was evaluated at week 8. The mean change from baseline in HAM-A total score was also used to discover the effects of celecoxib on ameliorating symptoms of anxiety (secondary outcome).

*Data analysis*

Due to small sample size, nonparametric tests were used. Two study groups were compared based on demographic variations. Baseline severity of depression and anxiety were compared using Mann-Whitney U-test. Additionally, Mann-Whitney U-test was used to compare depression and anxiety total scores at baseline, week 4 and week 8 between the two treatment groups. Within-group variables were analyzed by using Friedman test, a non-parametric alternative to the repeated-measure ANOVA. Wilcoxon Signed-Rank test was conducted to find the mean change which was significantly different from baseline for each group. Moreover, the Fisher exact test was used to analyze response rate, remission rate and adverse reactions. The p-value ≤ 0.05 was considered to be significant. A “complete case” (or “available case”) analysis was done in the present study. Data were analyzed by SPSS Statistics version 16.00.

## Results

All patients experienced first episode of major depression. There were no significant differences between patients who took celecoxib as an add-on or patients who only took sertraline, when demographic data and clinical features were compared (Table 1). The baseline severity and duration of depression did not significantly differ between the two groups. No significant differences were observed in mean sertraline dosage requirement between the two groups during the course of treatment (P = 0.63). 4 patients (2 patients in celecoxib and 2 patients in the placebo group) required to increase sertraline dosage to 100 mg/day.

**Table 1:** Demographic and clinical data of depressed patients who went on a clinical trial of celecoxib (200 mg/day) add-on treatment to sertraline (50-100 mg/day).

	**Sertraline ** **+ Celecoxib (n=14)**	**Sertraline ** **+ Placebo (n=9)**	**p-value**
Age, yr (Mean±SD)	34.7±7.3	36.2±12.7	NS
Weight, kg (Mean±SD)	62.42±13.0	69.1±8.7	NS
Height, m (Mean±SD)	1.5±5.9	1.5±14.4	NS
Marital status: N (%)			
Single Married Divorced or widowed	5 (35.7%) 8 (57.1%) 1 (7.1%)	2 (22.2%) 7 (77.8%) 0	NS
Education level: N(%)			
High school or less College or more	7(50%) 7 (50%)	5 (55.6%) 4 (44.4%)	NS
Drug naïve	All	All	NS
Onset of current episode, months (Mean±SD)	6±1.4	7.7±2.6	NS

**Figure 1:**
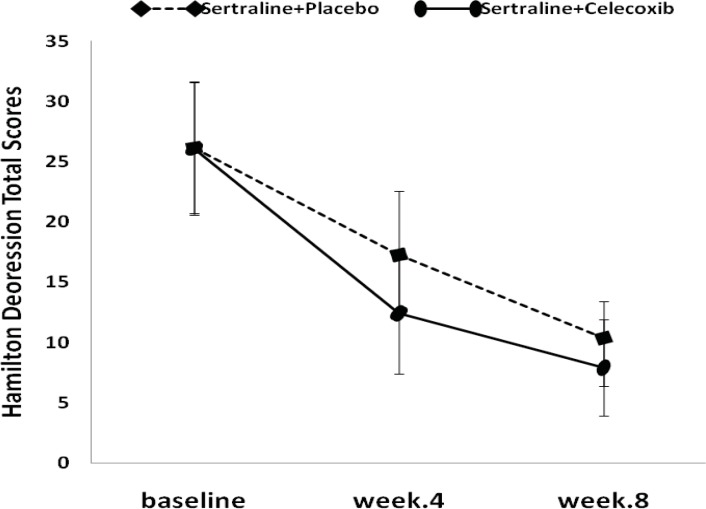
Hamilton Depression Rating Scale scores (Mean±SD) in major depressed patients who went on a clinical trial sertraline (50-100 mg/day) + placebo, versus sertraline (50-100 mg/day) + celecoxib (200 mg/day).

The results of the Friedman test indicated that HAM-D total score in both treatment groups decreased significantly from baseline (P<0.001, Figure 1).

The mean HAM-D score changes, from baseline to week 4, were -13.7±3.8 and -8.8±4.5 in the celecoxib and placebo groups, respectively. Celecoxib group showed significantly greater decrease in Hamilton score compared to placebo (P<0.05) at the end of week 4 (Figure 1, Table 2). 

* p-value ≤ 0.05, **p-value ≤ 0.01, ***p-value ≤ 0.001, p-values show significant improvement of HAMD scores during course of treatment in each group.

**† **p-value ≤ 0.05, Pvalue shows between group differences in HAMD scores during course of treatment.

**Table 2:** Hamilton Depression Rating Scale scores (Mean±SD) in both treatment groups

	**Before treatment**	**Week 4**	**Week 8**
Sertraline + Placebo	26.2±5.3	17.3±5.2	10.4±3.0
Sertraline + Celecoxib 200mg/day	26.1±5.5	12.4±5	7.9±4.0
p-value	NS^1^	0.021*	NS

1 Non-significant

* P≤ 0.05 considers significant.

**Figure 2:**
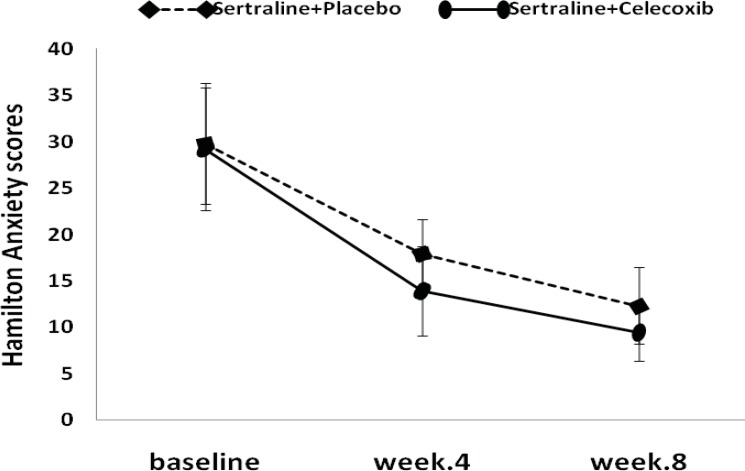
Hamilton Anxiety Rating Scale scores (Mean±SD) in major depressed patients who went on a clinical trial sertraline (50-100 mg/day) + placebo, versus sertraline (50-100 mg/day) + celecoxib (200 mg/day).

The mean HAM-A total score decreased from 29.2 at baseline to 13.9 at week 4 and from 29.2 at baseline to 17.8 at week 4, in the celecoxib and placebo groups, respectively. Differences between the two groups were statistically significant after 4 weeks of study (Figure 2, Table 3).

* p-value ≤ 0.05, **p-value ≤ 0.01, ***p-value ≤ 0.001, p-values show significant improvement of HAMA scores during course of treatment in each group.

† p-value ≤ 0.05, Pvalue shows between group differences in HAMA scores during course of treatment.

**Table 3:** Hamilton Anxiety Rating Scale scores (Mean±SD) in both treatment groups

	**Before treatment**	**Week 4**	**Week 8**
Sertraline + Placebo	29.7±6.4	17.8±3.7	12.3±4.2
Sertraline + celecoxib 200mg/day	29.2±6.5	13.9±4.8	9.4±3.0
P value	NS^1^	0.05*	NS

1 Non-significant

* P≤ 0.05 considers significant.

At the endpoint (week 8), the mean reduction in HAM-D scores from baseline was more in the treatment group (-18.3±3.4) compared to placebo group (-15.8±5.2), although it was not statistically significant (Figure 1, Table 2). Similar to depression score pattern after 8 weeks, the HAM-A total scores was not significantly different between the two groups; however, celecoxib group experienced greater reduction in anxiety scores (Figure 2, Table 3). 

Response rates (a ≥ 50% drop in HAM-D rating scores) were significantly greater (P< 0.05) in the celecoxib add-on group (57%) in comparison with placebo add-on group (11%) after 4 weeks. Surprisingly, although higher response rates (100%) were observed in the celecoxib group in comparison with the placebo group (77.7%) after 8 week treatment course, the difference did not reach the statistical significance (P=0.14) (Table 4). 

**Table 4:** response rate comparison

**Week 8**	**Week 4**	Response rate
7/9 (77.7%)	1/9 (11%)	Sertraline + Placebo
14/1 (100%)	8/1 (57%)	Sertraline + celecoxib 200mg/day
NS^1^	0.04*	P value

1 Non-significant

* P≤ 0.05 considers significant

After 8 weeks of treatment, remission rate (HAM-D ≤ 7) was significantly higher in the group receiving celecoxib (57%) versus placebo (11%) (P< 0.05). 

The incidence of adverse effects did not differ significantly in both treatment groups. The most common adverse events reported included agitation, anxiety, nausea, decreased appetite, headache, dyspepsia, and diarrhea. No major gastrointestinal problems (including bleeding, ulceration and perforation of stomach or intestine) and cardiovascular problems were observed in the patients. Indeed, that was not surprising, since previous studies which investigated the effects of higher doses of celecoxib (400 mg/day) together with an antidepressant did not report an increase in GI bleeding or other adverse effects associated with the use of NSAIDs (27,28). We used lower doses of Celecoxib (200 mg/day) and expected not to observe major side effects as well. In general, 7 patients discontinued the study during first three weeks. One patient in the celecoxib group discontinued treatment after 3 weeks because "she felt better" as expressed by patient herself. Also, 6 patients in the placebo group stopped taking their medication, one of them due to reported nausea, 2 of them because of decreased appetite and 3 of them because of feeling better.

## Discussion

Depressive disorder is the leading cause of disease burden for women aged 15–44 years in countries without regard to their income levels (34).

Inflammatory cytokines are potent inducers of Hypothalamic-pituitary-adrenal axis and are blamed to cause serotonin depletion (14, 35, 36). On the other hand, studies have found that depressed patients have increased level of cytokines which is seemingly correlated with the severity of depression and treatment response (4,37). Inflammatory cytokines such as TNF-*α*, IL-1*β *and IL-6 are the main inflammatory cytokines produced during CNS inflammation (38). Recently, researchers have shown an increased interest in understanding the effects of celecoxib in depression along with other psychiatric disorders (22-28)^.^

According to the present study, both groups showed notable improvement in disease symptoms during the trial. Nevertheless, improvement in celecoxib add-on group was remarkably greater at the end of week 4. Additionally, celecoxib adjunctive treatment resulted in higher response rate (5-fold more compared to placebo) after 4 weeks. These results may substantiate the assumption that celecoxib probably speed up the onset of therapeutic action of antidepressants and make the treatment more effective.

Depression tends to be a recurrent illness and remitted patients are less likely to be afflicted by relapse. Moreover, less than 30% to 40% of patients fully remitted after receiving adequate treatment. Accordingly, nowadays full remission of depressed patients is considered as preferred goal of treatment. In the present study, a 5-time increase in the number of remitted patients was observed in the celecoxib add-on group. 

Some published clinical trials examined the effect of celecoxib (400 mg/day) on previously treated depressed patients over 6 weeks of treatment (27,28). According to similar results significant reduction in HAM-D scores were observed after elapse of 6 weeks, while our findings showed beneficial effect of lower dosage of celecoxib after 4 weeks of treatment. It possibly lies in the fact that present study has been carried among drug-naïve and newly diagnosed major depressive disorder patients and the two above mentioned studies enrolled patients with past depressive episodes. As patients with past episodes are more prone to be non-responders, this may have affected the response rate in those studies. Eller *et al*. demonstrated that non-responders’ group had more previous depressive episodes and were treatment-experienced, thus not drug-naïve compared to the responders (37). Our findings strengthened the above mentioned idea as nearly 80% of patients in the placebo group and 100% in the celecoxib group were responders after 8 weeks. 

Moreover, the present study was done over a period of 8 weeks to observe the therapeutic effect of celecoxib within the time. According to the results, the beneficial effect of celecoxib on response rate and reduction of depressive symptoms appeared to be less important at week 8. The results confirmed previous findings that antidepressants like sertraline exert the optimal therapeutic effect after elapse of time. As other studies followed up the cases just in 6 weeks period of time, their results might not show the fading of positive effects of celecoxib along the time.

Also, this is the first clinical study which assessed the effect of celecoxib on depression at the 200 mg/day dosage**. **The reason for this dose selection lies in the fact that higher dosage of 400-800 mg/day did not show superior anti-inflammatory efficacy compared to lower dosage of 200 mg/day based on some clinical studies. Also, it was suggested that celecoxib may lose its anti-inflammatory efficacy at higher doses (39). Thus, we hypothesized that in this setting, using 200 mg/day dosage may be a more rational selection. All previously published clinical studies assessed celecoxib adjunctive effect in a fixed dose of 400 mg daily. Jian-You *et al.* showed that chronic treatment with 16 mg/Kg celecoxib reverses chronic unpredictable stress-induced depressive-like behavior through reducing COX-2 expression in rat brain. They indicated that the mentioned dosage is equivalent to 181 mg celecoxib for a 70 Kg man which confirms the rationale for selecting our administered dose (22). Based on our study results, celecoxib at the daily dose of 200 mg may have comparable efficacy to prior administered dosage of 400 mg/day. According to a recently published study with 6 weeks duration, celecoxib at the dose of 400 mg daily may show some antidepressant effects which is correlated with decreased levels of IL-6 at the end of study (40). It is important to note that cardiovascular (CV) side effects of celecoxib are dose-related and increase with taking 400 mg to 800 mg daily. These results demonstrated that there is no need to expose patients to the higher dosage of celecoxib which potentially leads to higher CV risks, even in short-term trials. 

## Conclusion

The results of the present study suggest that celecoxib adjunctive therapy at the dosage of 200 mg/day may accelerate the onset of therapeutic effect of sertraline and result in a better response and higher remission rate. However, while the optimal therapeutic effect of sertraline covers up the beneficial effect of celecoxib augmentation within 8 weeks, celecoxib may be considered as an option to speed up the onset of action of antidepressants such as sertraline during acute phase of treatment.

As our patient population was homogeneous and gender specified, the results may not be extrapolated to the entire population due to inter-gender differences. Moreover, as we selected fixed dosage of celecoxib (200 mg/day), the lower or higher doses may have shown different results.
